# Extraction of thorium from aqueous system using citric acid modified corn cob: characterization, kinetics, thermodynamics and adsorption mechanism

**DOI:** 10.1038/s41598-026-47530-6

**Published:** 2026-05-07

**Authors:** Reda M. Attia

**Affiliations:** Nuclear Materials Authority, P.O. Box 530, El-Maadi, Cairo, Egypt

**Keywords:** Corn cob, Citric acid, Thorium, Adsorption, Elution, Kinetics, Chemistry, Environmental sciences, Materials science

## Abstract

This study investigates the potential of low-cost, eco-friendly, chemically modified corn cob treated with citric acid as an efficient adsorbent for the removal of Th^4+^ ions from aqueous solution. The structural, morphological, and surface characteristics of the natural and modified adsorbent were investigated by FTIR, XRD, SEM, EDX, BET, and XPS techniques. Citric acid modification significantly enhanced the surface area, porosity, and the number of oxygen-containing functional groups, particularly carboxyl groups, thereby improving the adsorption performance. The effects of pH, contact time, adsorbent dose, initial Th^4+^ concentration, and temperature were examined. The maximum adsorption efficiency reached 90.2% at pH 4, with equilibrium achieved in 25 min at room temperature, and an optimal adsorbent dose of 1 g L^−1^. The adsorption equilibrium data were well described by the Langmuir isotherm model, indicating monolayer adsorption with a maximum capacity of 196.08 mg g^−1^. The kinetic studies showed that the process followed the pseudo-second-order model. Thermodynamic parameters (ΔG = − 19.08 to − 22.73 kJ mol^−1^, ΔH = + 17.23 kJ mol^−1^, ΔS = 121.83 J mol^−1^K^−1^) indicate that the process is spontaneous, endothermic, and accompanied by increased randomness at the solid–liquid interface. Desorption using 0.05 M HNO_3_ achieved an efficiency of 94%, which slightly decreased to 88% after five successive cycles.

## 1. Introduction

Thorium is found in a wide range of minerals, it is usually present as oxides, silicates, phosphates, and in smaller amounts in other minerals. The main source of thorium is the rare earth phosphate monazite, which typically contains less than 10% thorium. Thorium can be found in various minerals, including monazite, xenotime, zircon, and ilmenite^1^. Other and commercially important minerals are ilmenite, rutile, and magnetite. Monazite can be separated, and if thorium is needed, it can be extracted as a byproduct. The scientific and commercial value of thorium stems from its many applications in fields like optics, radios, aeronautics, aerospace, metallurgy, chemical and nuclear industries, materials science, and nuclear medicine^2^.

Several techniques have been employed for the extraction of thorium, including precipitation^3^, solvent extraction^4^, and adsorption^2,5–10^. Other methods used include ion exchange, electrowinning, crystallization, and membrane separation.

Various types of agricultural waste materials have been identified in studies, including cotton stalks, corncobs, stone fruits, date seeds, rice straw, and wood^11^. These materials are primarily composed of cellulose, hemicelluloses, and lignin, along with functional groups like hydroxyl, aldehyde, carbonyl, carboxyl, and phenolic groups, which can form stable complexes with metal ions^11^. However, a significant portion of the world’s agricultural resources are wasted and not recycled^12,13^. Agricultural waste materials can be used to create effective biosorbents that remove inorganic and organic pollutants from wastewater due to their abundance, environmental safety, renewability, and low cost^14,15^. Although these biosorbents have a lower sorption capacity than chemical synthetic adsorbents^11,15,16^, their adsorption efficiency can be improved through physical and chemical treatments. Chemical treatments typically use reagents such as bases like sodium hydroxide, calcium hydroxide, and sodium carbonate; mineral acids like nitric acid, hydrochloric acid, and sulfuric acid; organic acids like citric acid, oxalic acid, and tartaric acid; and organic compounds like formaldehyde and methanol, along with hydrogen peroxide as an oxidizing agent^11^. The acidification process, which is part of wet oxidation, removes mineral impurities from agricultural waste and enhances the adsorbent’s acidic behavior and hydrophilic nature^17^. Additionally, treating the adsorbent with acids like H_3_PO_4_, HCl, HNO_3_, and H_2_SO_4_ alters functional groups, modifies surface area and porosity characteristics, to improve the adsorbent’s adsorption performance^18^.

According to Arab Finance, Egypt produced 6.3 million tons of corn in 2023^19^, and according to the United States Department of Agriculture (USDA, 2024)^20^, For marketing year 2024/25, Egypt’s corn production is forecast at about 7.0 million tons. The high demand for corn grains generates a lot of corn cobs, which are a major source of agricultural waste and contribute to environmental pollution. Typically, these cobs are discarded or burned to produce low-quality fuels, releasing carbon dioxide and adding to global warming^21^. There is a large ratio between corn grain and corn cob (100:18), leading to the accumulation of significant amounts of unused corn cobs worldwide^13^. Corn cobs mainly consist of 39.1% cellulose, 42.1% hemicellulose, 9.1% lignin, 1.7% protein, and 1.2% ash^21^. Since they lack nutrients and other useful vitamins, corn cobs are not widely used; the most common practice is burning them for heat energy^22^. Corn cobs have been used as natural and effective adsorbents for heavy metals from aqueous solution. For example, Arul, et al. used magnetic corn cobs to remove Pb (II) and Cd (II) ions, with maximum adsorption capacities of 127 mg g^−1^ and 100 mg g^−1^, respectively^23^. Melese, et al. reported maximum removal efficiencies for Pb, Cd, Cr, and Cu of 88.27%, 85.91%, 72.84%, and 77.94%, respectively, at 100 mg L^−1^ using natural corn cobs^24^. Ajeng et al. explored the adsorption of Cu and Zn from water, achieving 89.3% and 89.2% efficiency, with maximum capacities of 75.76 mg g^−1^ and 63.93 mg g^−1^, respectively, using magnetically modified corn cobs^25^. The maximum removal rates for Cd, Ni, and Zn with activated carbon from corn cobs were 100%, 100%, and 97.2%, respectively^26^. The adsorption capacity of natural corncobs for Cd^2+^ increased from 4.7 to 19.3 mg g^−1^ after treatment with nitric acid^27^. Altun and Pehlivan^28^ and Monroy-Figueroa et al.^15^ found that chemical treatment with citric acid is more effective than other modifications because it strengthens the waste and adds acidic groups, improving adsorption for metals. Generally, chemical modifications remove lignin and hemicellulose, reduce cellulose crystallinity, and increase porosity or surface area, leading to higher adsorption capacities compared to unmodified waste. Citric acid, a low-cost organic polycarboxylic acid with three carboxyl groups, reacts with cellulose hydroxyl groups via esterification^29^. It also forms strong complexes with thorium as Th^4+^ citrate complexes^30^. Leyva-Ramos et al. reported that treating corncobs with citric acid increased surface area and oxygen content by introducing oxygenated groups, especially acidic sites like carboxylic, phenolic, and lactonic groups^27^. This lowered the pH_ZPC_, increasing adsorption capacity compared to untreated corncobs. Various natural adsorbents have been used to remove Th^4+^ from aqueous solution. Abbas showed that banana peel effectively removes thorium from wastewater, with a removal efficiency of 95.34%^31^. Ginkgo leaves also selectively adsorb Th^4+^ over lanthanides, with a maximum capacity of 103.8 mg g^−1^^2^. Khamseh and Ghorbanian studied Th^4+^ adsorption using orange peel in a fixed-bed column; the highest sorption capacity was 87.7 mg g^−1^ with a sorbent size of 0.4–0.8 mm^32^. Varala et al. reported that rice husk could adsorb 15.95 mg g^−1^ of Th^4+^^33^. Alkaline-modified aloe vera showed an impressive capacity of 170 mg g^−1^ for Th^4+^^7^. Citric acid-modified Lemna minor achieved a maximum of 162.5 mg g^−1^ for Th^4+^^34^. Date seed effectively removes thorium ions from acidic solutions, with a capacity of 43 mg/g^35^. Pomegranate peel was also tested as a biosorbent and reached a sorption capacity of 80 mg g^−1^ for thorium^8^.

The aim of this study is to remove Th^4+^ from aqueous solution using a natural, eco-friendly modified corn cob treated with citric acid. Natural and modified corn cob are characterized using infrared spectroscopy, scanning electron microscopy, X-ray diffraction and X-ray photoelectron spectroscopy. The effects of various parameters, including, pH, the amount of adsorbent used, shaking time, the initial Th^4+^ concentration, and temperature, on the adsorption process are investigated. In addition, the desorption of loaded Th^4+^ from the modified corn cob is studied. Furthermore, the adsorption isotherms, kinetics, and thermodynamic behavior of Th^4+^ are evaluated. The novelty of this work lies in the use of citric acid modified corn cob as a low-cost and sustainable biosorbent for the efficient removal of Th^4+^ and the investigation of its adsorption mechanism.

## Experimental

### Chemical and analytical procedure

For this work, all chemicals and reagents used were of analytical grade. A standard solution of Th^4+^ with various concentrations was prepared from stock solutions of 1000 mg L^−1^ Th (NO_3_)_4_ by diluting them with distilled water. The pH of the standard solution was adjusted using 0.1 M solutions of HCl and NaOH. Chemical analysis of major components was carried out using the rapid silicate analytical procedure^35^. Trace elements were characterized through X-ray fluorescence technique (XRF) using Philips Unique II unit equipped with an automatic sample changer PW 1510 (30 position), interfaced with a computer system employing the X-40 program for spectrometry at the Nuclear Materials Authority Laboratories (NMA Lab). A single-beam UV-VIS Metertech SP-8001 spectrophotometer was used to measure the concentration of Th^4+^ using the thoron (I) method, which forms a colored complex with Th^4+^^36^. Uranium was quantified via the oxidimetric titration method with ammonium metavanadate^37^.

### Modification and characterization of corn cob (CC)

Researcher collected corn cobs (CC) from farmland in Egypt. The CC was first crushed, ground, and sieved to a particle size of 0.125 mm. The CC was then washed several times with distilled water and dried. To enhance the adsorption properties of the CC, chemical modifications using citric acid was performed. Following the methods outlined by Leyva-Ramos et al.^38^ and Monroy-Figueroa et al.^15^ through mixing 5 g of CC with 25 mL of citric acid solutions at different concentrations (0.5 M to 2 M) and stirred the mixture for 2 h at 60 °C and 150 rpm. After cooling, the adsorbent was filtered, dried at 50 °C for 24 h, and then exposed to heat at 120 °C for 2 h. Lastly, the modified corn cobs (MCC) were washed with deionized water until the pH stabilized, and they were dried at 50 °C for 24 h.

The Fourier-transform infrared (FTIR) spectrum was used to analyze the functional groups of adsorbents responsible for Th^4+^ ion adsorption. The adsorbent was mixed with KBr, ground, and pressed into a disc using a special press to form a standard KBr pellet. The infrared spectra of this disc were captured using Shimadzu FT- IR 8101 PC spectrophotometers with a Pye Unicam SP 3300. the surface properties of the adsorbent. X- ray diffraction (XRD) was used to determine the crystal structure of the adsorbent using a Smart Lab X-Ray Diffractometer (RIGAKU, Japan). The surface morphology of the adsorbent was characterized before and after modification using Scanning Electron Microscopy (SEM) with model XL 30, supported by an energy dispersive X- ray (EDX) unit. EDX confirmed the modification processes and determined the dispersion and concentration of thorium before and after adsorption. The specific surface area and pore characteristics of natural and modified corn cob were determined using the Brunauer–Emmett–Teller (BET) method with a Quantachrome Nova LX2 surface area analyzer based on N_2_ adsorption–desorption measurements. X-ray photoelectron spectroscopy (XPS) was used to analyze MCC and thorium-loaded MCC using a Thermo Fisher Scientific instrument (Waltham, MA, USA). The point of zero charge (pHpzc) of the adsorbent was defined as the pH at which the positive and negative charges on its surface are balanced, resulting in a neutral net charge^39^. At this pH, the adsorption process is primarily governed by metal ion diffusion into micro- and macro- pores, where the electrostatic forces do not play significant role^40^. Approximately 0.1 g of MCC samples was mixed with 50 mL of 0.1 M NaCl solution, pre- adjusted to initial pH values ranging from 1 to 10 using 0. 1 M HCl or NaOH. The suspensions were continuously agitated at 150 rpm and maintained at room temperature for 24 h to ensure equilibrium pH conditions. Afterward, the suspensions were filtered, and their final pH was measured^38^. A graph plotting the change in pH (pH_i_- pH_f_) against the initial pH (pH_i_) was constructed, and the point where the graph intersects the pH axis- where pH is zero- was identified as the pH_pzc_ of MCC.

### Adsorption process

Various parameters were studied to maximize the adsorption capacity of MCC for Th^4+^ using a batch technique. These included pH, initial Th^4+^ concentration, contact time, and the impact of temperature. A mixture of approximately 0.02 g of MCC and 20 mL of Th^4+^ standard solution at a specific concentration was shaken in a glass vial on a thermostatic shaker water bath at 150 rpm for a set time. The filtrate was then removed from the adsorbent solution using filter paper, and the Th^4+^ concentration was measured. The adsorption capacity (q_e_, mg g^−1^) and adsorption efficiency (E, %) are calculated using the following equations: Eqs. (1,2):

1 $${{\mathrm{q}}_{\mathrm{e}}}=\left( {{{\mathrm{C}}_0} - {\mathrm{Ce}}} \right) \times \frac{{\mathrm{V}}}{{\mathrm{M}}}$$

2 $$\mathrm{E}\%=\frac{{{{\mathrm{C}}_0} - {\mathrm{Ce}}}}{{{{\mathrm{c}}_0}}} \times 100$$

where C₀ and Cₑ are the initial and equilibrium concentrations of Th^4+^ in milligrams per liter, respectively; M is the amount of adsorbent in grams, and V is the volume of the Th^4+^ solution in liters.

### Desorption and recycling process

The loaded MCC adsorbent, prepared under the optimized adsorption conditions, was subjected to desorption studies using different eluting agents, namely HCl, HNO_3_, H_2_SO_4_, and EDTA, to evaluate the desorption efficiency as a function of eluent type, concentration, and contact time. For this purpose, 0.005 g of loaded MCC was mixed with 5 mL of the eluting solution at various concentrations and times. The Th^4+^ concentration in the filtered solution was then analyzed. The elution efficiency of thorium was calculated using the following equation:

3 $$elution (\%)= 1-\left( {\frac{{{\mathrm{Th}}_{{{\mathrm{ads}}}}^{{4+}} - {\mathrm{Th}}_{{{\mathrm{des}}}}^{{4+}}}}{{{\mathrm{Th}}_{{{\mathrm{ads}}}}^{{4+}}}}} \right) \times 100$$

Where ${\mathrm{Th}}_{{{\mathrm{ads}}}}^{{4+}}$ and ${\mathrm{Th}}_{{{\mathrm{des}}}}^{{4+}}$ were the amount of adsorbed and desorbed thorium (mg L^−1^).

Following desorption processes, the MCC was rinsed with distilled water and then reused in Th^4+^ adsorption experiments. As part of the adsorption-desorption study, the adsorbents were recycled five times to evaluate their recyclability and stability. All experiments were performed in triplicate to ensure accuracy and reproducibility, and the mean values were reported.

### Application

A geological representative sample of cataclastic rock was collected from the Abu Rusheid area in the South Eastern Desert, Egypt. The obtained sample was carefully crushed, ground to approximately 75 μm, then quartered to obtain a representative sample and dried. The chemical analysis of major and trace elements illustrated in (Table 1). About 5 g of this sample was subjected to nitric acid leaching under condition of 5 M HNO_3_, with S/L ratio of 1/5 for 1 h leaching time at 90 °C temperature as reported in Afifi et al.^41^. After leaching process, the solution cooled, filtered. The solid residue was washed with distilled water; the combined filtrate was then made up to the desired volume with distilled water. For adsorption experiment, take 20 ml of leach liquor, adjust pH firstly at 3.5 where a reddish-brown precipitate of ferric hydroxide removed. Then apply the optimum adsorption parameters upon filtrate using suitable amount of MCC. The concentration of thorium in the solution before and after adsorption was determined using the thoron (I) spectrophotometric method. The concentrations of interfering elements were analyzed using ICP-OES (Inductively Coupled Plasma Optical Emission Spectrometer, Prism ICP, Teledyne Leeman Labs., Hudson, NH, USA).

**Table 1:** Chemical analysis of the studied sample.

Major oxides	Wt., %	Trace elements	mg kg^−1^
SiO_2_	67.0	Th	1098.6
Al_2_O_3_	13.86	U	385.6
TiO_2_	0.43	ƩREEs	1560
Fe_2_O_3_	5.80	Cu	85
CaO	2.60	Zn	1053
MgO	1.15	Pb	118
Na_2_O	5.15	Zr	452
K_2_O	1.84	Ba	204
P_2_O_5_	0.41	Nb	45
L O I ^*^(1000^°^C)	1.71		
Total	99.95		

## Result and discussion

### Characterization of chemical modified corncob (MCC)

#### FTIR analysis

**Fig. 1:**
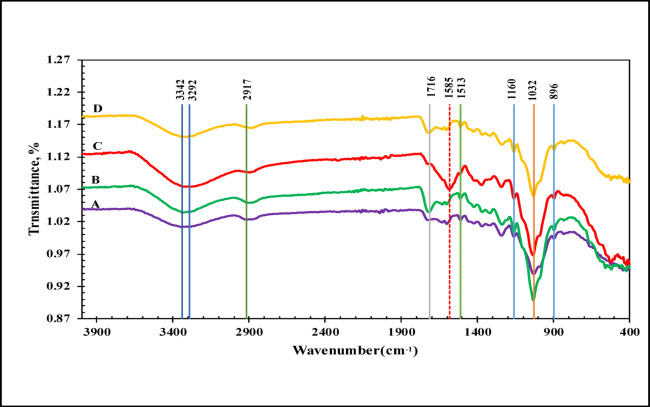
FTIR spectra of (A) CC, (B) MCC, (C) MCC Loaded with Th^4+^ and (D) After desorption.

FTIR spectra of the original CC, modified with citric acid MCC, Th^4+^ -loaded MCC, and after thorium desorption are shown in Fig. 1. The infrared spectra of cellulose in all samples exhibit a strong, broad band from 3292 to 3342 cm^–1^, which is attributed to the stretching of OH groups related to alcohols and phenols in cellulose and lignin^2,15,24^. Peaks observed from 2889 to 2917 cm^–1^ are associated with symmetric and asymmetric C–H stretching vibrations typical of cellulose^2,42^. A peak at 1513 cm^–1^ is attributed to C=C in aromatic rings^15^. The band at 1160 cm^−1^ corresponds to C–O–C asymmetrical bridge stretching^43^. The peaks at 1032 cm^–1^ is characteristic of C–O stretching in primary hydroxyl groups associated with cellulose structure^34^. The absorption band at 896 cm^−1^ is attributed to β-(1→4) glycosidic linkages between glucose monomers^44^. The reaction between citric acid and corncob increases the intensity of the carbonyl band at 1716 cm^−1^, due to esterification and an increase in COO groups in the modified corncob^38,45^. This indicates the successful incorporation of citric acid into the cellulose structure of CC^43,46–48^. The FTIR spectrum of MCC loaded with Th^4+^ shows a significant shift of the peak from 1716 to 1585 cm^–1^, reflecting changes in the carboxylate stretching frequency upon binding with Th^4+^. This reduction suggests that the carbonyl oxygen’s is involved in coordination with Th^4+^. Additionally, changes in the peak from 3335 to 3292 cm^–1^ indicate hydroxyl groups are involved in adsorption processes^2,7,34^. As shown in (Fig. 1), these changes in stretching frequencies revert back to 1716 and 3337 cm^–1^ after Th^4+^ desorption. Moreover, the intensity of the absorption bands observed in the FTIR spectrum of MCC increases after Th^4+^ adsorption.

**Fig. 2:**
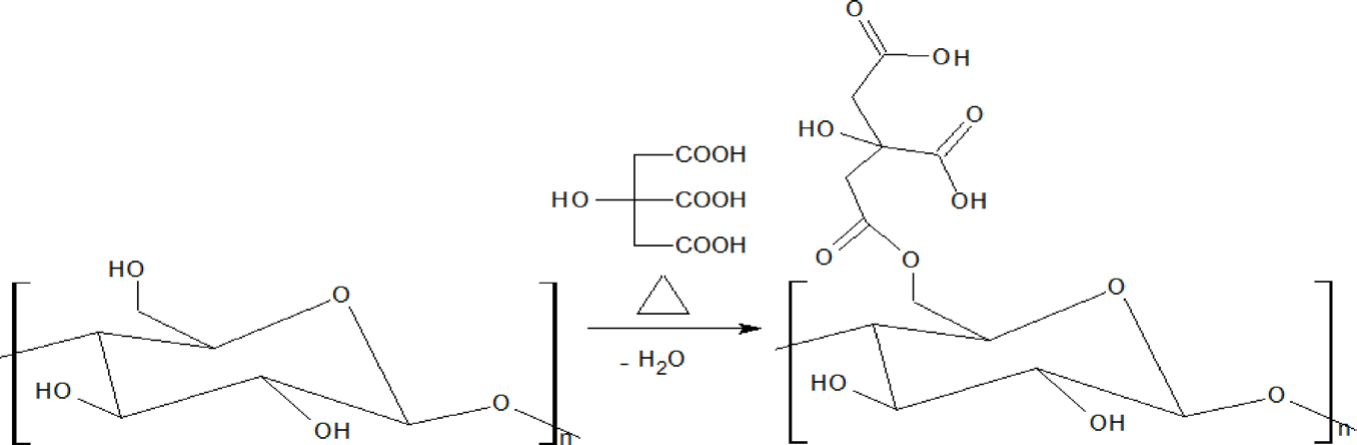
Reaction of corn cob with citric acid.

As illustrated in Fig. 2, citric acid undergoes esterification with the hydroxyl groups present in cellulose and lignin components of corncob, leading to the formation of modified corn cob (MCC) enriched with acidic functional sites. This modification introduces additional hydroxylic and carboxylic groups, thereby enhancing the material’s chemical reactivity and adsorption capability. The mechanism involves the initial formation of a reactive citric anhydride intermediate, followed by nucleophilic attack of the C6–OH groups of the corncob matrix on the anhydride carbonyl carbon, resulting in the formation of stable ester linkages (–COO–). This process proceeds via a condensation reaction pathway, accompanied by the elimination of a water molecule as a byproduct^49,50^.

#### XRD characterization

**Fig. 3:**
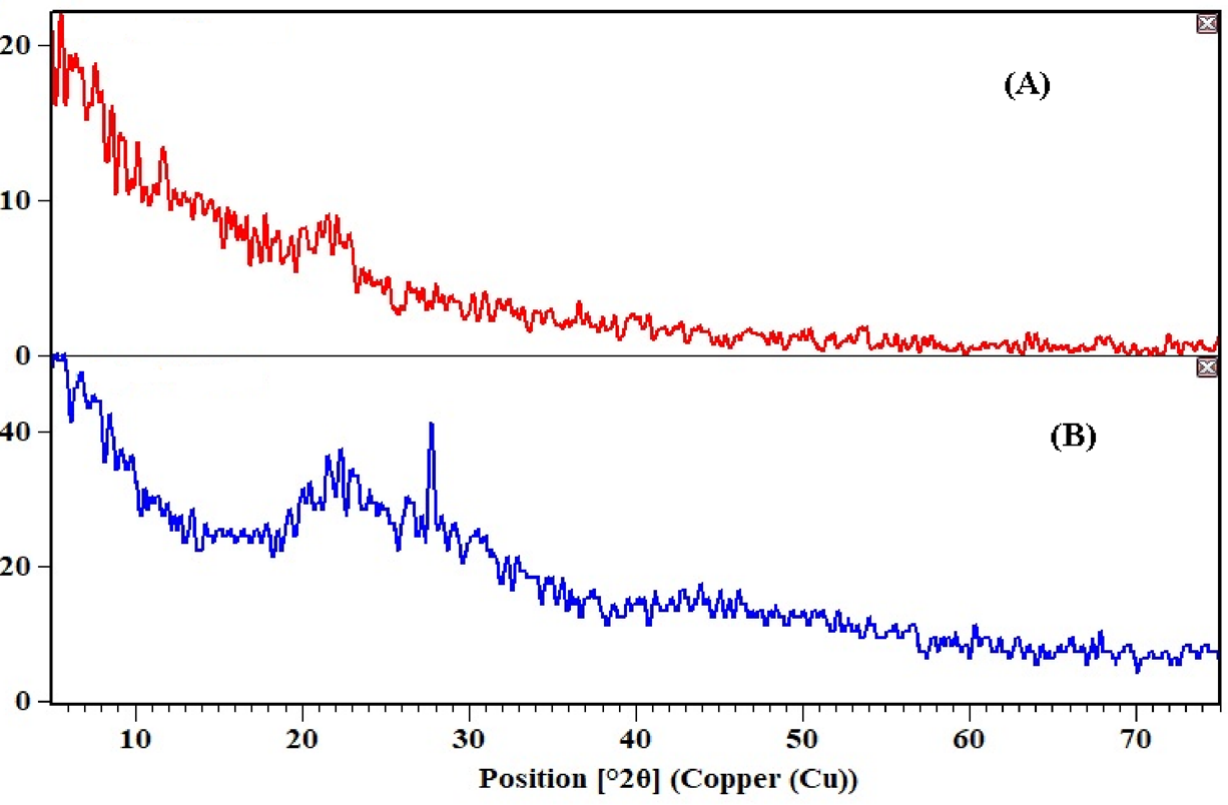
X-ray diffraction patterns MCC (**A**) before loaded and (**B**) after Th loaded.

The X-ray diffraction (XRD) patterns of MCC and Th^4+^-loaded MCC are presented in Fig. 3. The diffraction profile of MCC reveals a predominantly amorphous structure, as evidenced by the absence of well-defined crystalline peaks. Upon loading with Th^4+^ ions, distinct diffraction peaks emerge at 2θ values of 27.79° and 45.13°, corresponding to d-spacing values of 3.21 and 1.69 Å, respectively. These reflections are consistent with the standard PDF2 card No. 42-1462, confirming the formation of crystalline thorianite (synthetic ThO_2_) on the MCC surface.

#### SEM and EDX analysis

**Fig. 4:**
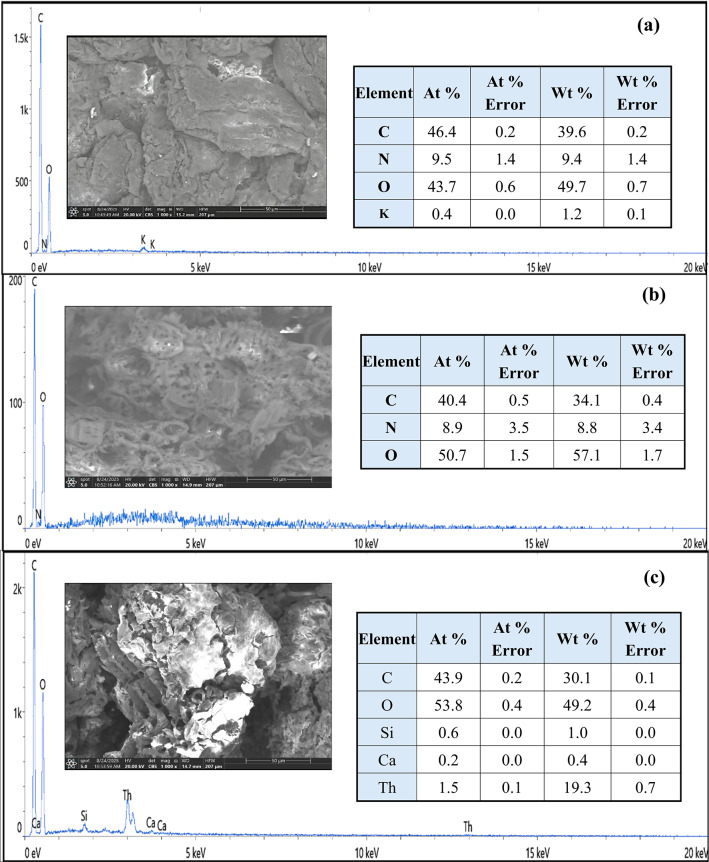
SEM-EDAX of (**a**) CC, (**b**) MCC, (**c**) MCC loaded with Th^4+^at 1000× magnification.

The SEM–EDX results presented in Fig. 4 provide detailed insight into the morphological evolution and elemental composition of CC, MCC, and Th^4+^-loaded MCC. The SEM image of CC (Fig. 4a) reveals a relatively compact, rough, and heterogeneous surface with limited porosity, reflecting the intact lignocellulosic structure. The surface appears partially smooth with few accessible adsorption sites. The corresponding EDX analysis indicates that CC is mainly composed of carbon (46.4 at%) and oxygen (43.7 at%); reflecting the cellulose, hemicellulose, and lignin matrix, with minor nitrogen (9.5 at%) and potassium (0.4 at%), corresponding to natural organic and inherent mineral ash. After chemical modification (Fig. 4b), MCC exhibits a significantly rougher and more porous morphology with developed channels and cavities, confirming structural disruption by citric acid treatment. This is supported by EDX results, which show a decrease in carbon (40.4 at%) and an increase in oxygen content (50.7 at%), indicating the successful incorporation of oxygenated functional groups such as carboxyl and hydroxyl groups. The slight nitrogen reduction (8.9 at%) confirms chemical restructuring and native component removal, evidencing successful functionalization critical for enhanced adsorption. In Fig. 4c, the Th^4+^-loaded MCC surface is covered with bright, dense agglomerates, suggesting effective metal deposition and partial blockage of pores^51^. The EDX spectrum confirms thorium adsorption, with Th detected at 1.5 at% (19.3 wt%), alongside minor Si (0.6 at%) and Ca (0.2 at%) impurities. The relatively high oxygen content (53.8 at%) further supports the involvement of oxygen-containing functional groups in Th^4+^ binding^52^. These results confirm successful modification and efficient thorium uptake via surface complexation mechanisms.

#### BET analysis

The BET results indicate that the MCC surface area is almost seven times larger than that of unmodified CC as presented in Table 2. Treating corn cob with 1 M citric acid greatly improved its textural properties. The BET surface area rose from 4.88 m^2^ g^−1^ to 35.89 m^2^ g^−1^, showing a significant increase in surface accessibility. The cumulative pore volume also increased from 0.0246 cm³ g^−1^ to 0.1845 cm³ g^−1^. Furthermore, the average particle radius decreased from around 279.47 nm to 37.992 nm, suggesting that citric acid caused particle fragmentation or surface etching. The pore size distribution shifted from mainly microporous (< 2 nm) at 1.6 nm in untreated corncob to mesoporous at 2.7 nm after citric acid treatment^52,53^. This change is beneficial for adsorption in aqueous solutions, as mesopores (2–50 nm) provide accessible adsorption sites and act as channels for hydrated metal ions, which often cannot enter micropores (< 2 nm)^52,53^. These results confirm that citric acid effectively activates the corn cob surface, increasing its porosity and surface area, and making it more suitable for adsorption applications.

**Table 2:** The textural properties of adsorbent materials.

Adsorbent materials	BET surface area (m^2^ g^−1^)	Average particle radius (nm)	Total pore volume (cm^3^ g^−1^)	Pore size (nm)
CC	4.87928	279.47	0.0246	1.6
MCC	35.8926	37.992	0.1845	2.7

#### X-ray photoelectron spectroscopy

**Fig. 5:**
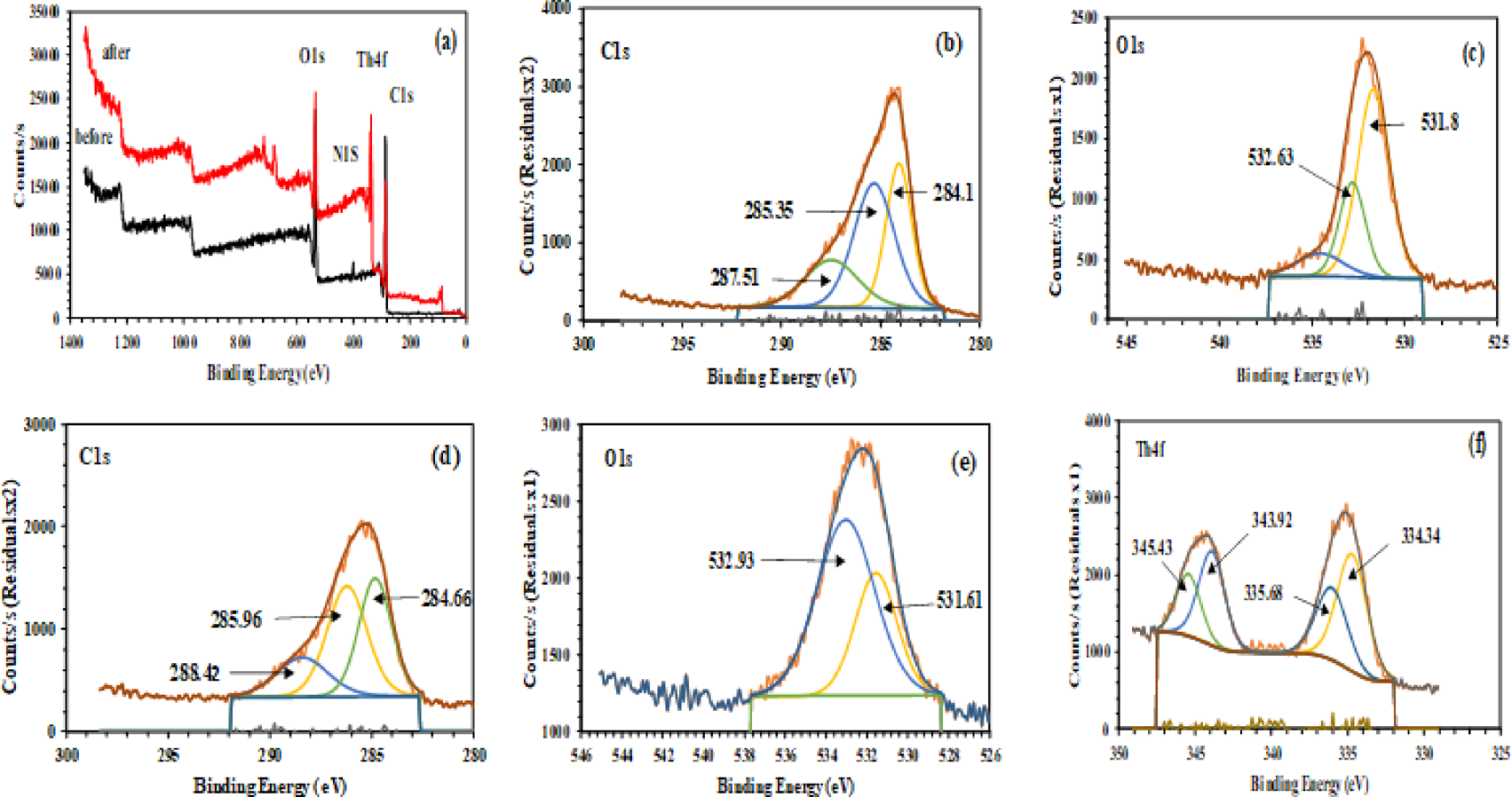
(**a**) Full-scan XPS, (**b**, **c**) XPS (C 1*s*, O1*s*) spectra of MCC, (**d**, **e**) XPS (C 1*s*, O1*s*) spectra of Th^4+^-loaded MCC, (**f**), HR-XPS (Th 4*f*) spectrum.

The XPS analysis determines electron binding energies, which depend on valence charge distribution, providing critical insights into the chemical environment and oxidation states of elements^52,53^. The survey spectrum of MCC (Fig. 5a) shows that the material is mainly composed of carbon and oxygen, with dominant C1s (~ 285.83 eV, 71.98%) and O 1s (~ 533.05 eV, 24.53%) peaks attributed to the cellulose backbone and oxygen-containing functional groups, while trace N1s peak (~ 400.86 eV, 3.49%) confirms amine functionalities^52,53^. The high-resolution C 1s spectrum of MCC before adsorption (Fig. 5b) was deconvoluted into three main peaks at 284.1 eV (C–C/C–H), 285.35 eV (C–O, hydroxyl or C–O–C ether groups), and 287.51 eV (O–C=O, carboxyl groups)^54,55^. Figure 5c shows the high-resolution O1s peaks of MCC before Th^4+^ adsorption. The peak observed at 531.8 eV (C=O/COOH) corresponds to double-bonded oxygen atoms in carbonyl groups, while the peak at 532.63 eV (C–O/OH) relates to single-bonded oxygen in hydroxyl and/or ether groups. After Th^4+^ adsorption, noticeable shifts in the binding energies of both C1*s* and O1*s* peaks toward higher values are observed, indicating that the oxygen-containing functional groups participate in coordination with Th^4+^, consistent with the formation of Th–O bonds (Fig. 5d,e). The decrease in carboxyl peak intensity, along with the shift in O1*s* peaks, suggests strong interaction and chelation between Th^4+^ ions and surface hydroxyl and carboxyl groups, confirming a chemisorption mechanism. The appearance of distinct Th 4f peaks after adsorption confirms the successful loading of thorium onto the MCC surface. The high-resolution Th 4f spectrum (Fig. 5f) exhibits two characteristic doublet peaks at 334.34–335.68 eV (Th 4f_7/2_) and 343.92–345.43 eV (Th 4f_5/2_), with a spin–orbit splitting (ΔBE) of approximately 9.58 eV confirming the presence of Th^4+^ in an oxide-like coordination environment^55,56^. The intensity ratio of Th 4f_7/2_ to Th 4f_5/2_ (~ 4:3 consistent with theoretical spin–orbit splitting), together with the high adsorption capacity (196.08 mg g^−1^)^57^ further confirms that thorium is successfully adsorbed onto the MCC.

#### pH_PZC_

**Fig. 6:**
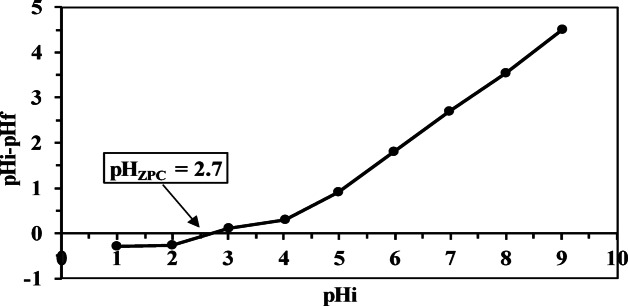
Point of zero charge of MCC.

Figuring out the pH_PZC_ value is crucial to find the optimal pH where anions or cations in solution are adsorbed most efficiently^58^. The graph in (Fig. 6) shows how the charge distribution on the MCC surface changes within a pH range of 1.0 to 10.0. The pH_PZC_ of MCC is 2.7, meaning the MCC surface is positive at pH levels below this value. As a result, the adsorption of Th ions expected to be more favorable at pH levels above 2.7 due to electrostatic attraction forces between Th^4+^ ions and the negatively charged MCC surface.

### Optimization of adsorption process using chemical modified MCC

#### Effect of citric acid concentration

**Fig. 7:**
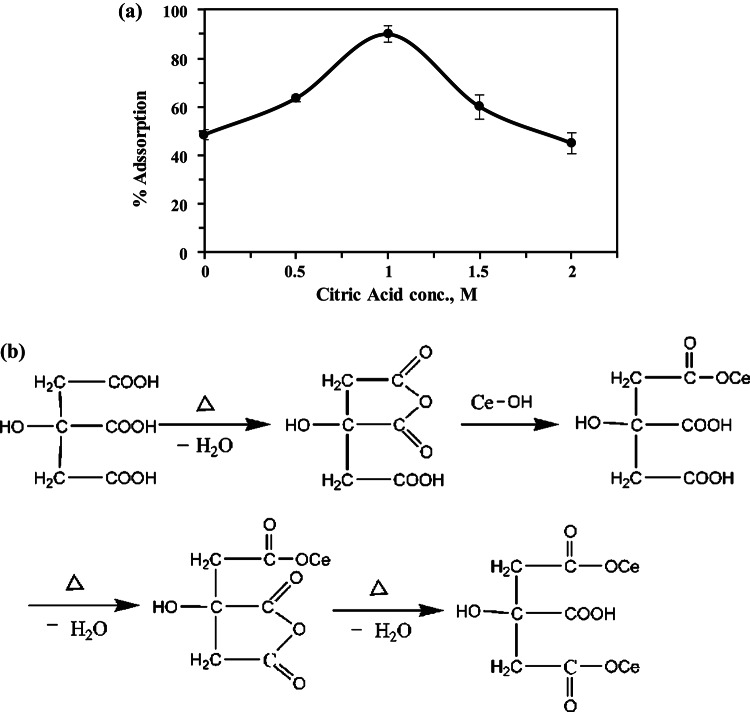
(**a**) Effect of Citric acid concentration upon adsorption efficiency of Th^4+^. (**b**) Reaction of corn cob with citric acid.

The influence of citric acid concentration during chemical treatment of CC has been studied from 0.5 M to 2 M. The adsorption experiment performed using fixed adsorption conditions: 100 mg L^−1^ Th^4+^ concentration, a 1:1 MCC-to-solution ratio, pH 4, for 30 min shaking time at room temperature. The results, shown in (Fig. 7a), indicate that at absence of citric acid the adsorption efficiency of Th^4+^ is 48.43%, increasing the citric acid concentration from 0.5 M to 1 M significantly boosted the adsorption efficiency of Th^4+^, from 63.5% to 90% respectively. This modification of CC is thought to occur according to a proposed reaction mechanism between citric acid and CC. Initially, citric acid is heated and dehydrates into reactive citric anhydride, which then reacts with the hydroxyl groups of cellulose and lignin to form an ester, introducing carboxylic groups to CC^49^. As the citric acid concentration rises, each molecule adds two more carboxylic sites, increasing the concentration of carboxylic and hydroxyl groups, peaking at 1 M. However, further increases to 1.5 M and 2 M led to a decrease in Th^4+^ adsorption efficiency. This decline is attributed to a reduction in available acidic sites due to cross-linking among the carboxylic groups attached to the corncob, which forms esters and decreases the number of free carboxylic sites (Fig. 7b), as explained by Leyva-Ramos et al.^38^. Higher acidity levels can also cause cellulose to decompose^45,59^. Therefore, 1 M was chosen as the optimal concentration.

#### Effect of initial pH

Solution pH critically influences adsorbent surface charge and functional groups through protonation–deprotonation equilibria, thereby governing adsorption behavior. Additionally, pH affects metal ion speciation in solution, altering their interaction with active sites and significantly impacting overall adsorption efficiency^52,53,60^. According to Leyva-Ramos et al. the point of zero charge (PZC) for natural (CC) is 5^38^. In this study, the PZC for modified MCC treated with 1 M citric acid was 2.7 (Fig. 6), which falls within the range reported by Leyva-Ramos et al. of 2.4^38^. As shown earlier, modifying CC with citric acid (CA) increases the number of acidic sites, resulting in a lower PZC value. This means that at pH values below 2.7, the MCC surface carries a positive charge, while at pH values above 2.7, the MCC surface becomes negatively charged, making it suitable for adsorbing Th^4+^. The positive and negative charges on the MCC surface are created by the protonation and deprotonation of carboxylic and hydroxylic groups, respectively, as illustrated by the following equation.

4 $${\mathrm{MCC}} {-} {\mathrm{COOH}}+{{\mathrm{H}}^+} \to {\mathrm{MCC}} {-} {\mathrm{COOH}}_{2}^{+}$$

5 $${\mathrm{MCC}}{-}{\mathrm{OH}}+{{\mathrm{H}}^+} \to {\mathrm{MCC}} {-} {\mathrm{OH}}_{2}^{+}$$

6 $${\mathrm{MCC}} {-} {\mathrm{COOH}} \to {\mathrm{MCC}} {-} {\mathrm{CO}}{{\mathrm{O}}^ - }+{{\mathrm{H}}^+}$$

7 $${\mathrm{MCC}} {-} {\mathrm{OH}} \to {\mathrm{MCC}} {-} {{\mathrm{O}}^ - }+{{\mathrm{H}}^+}$$

**Fig. 8:**
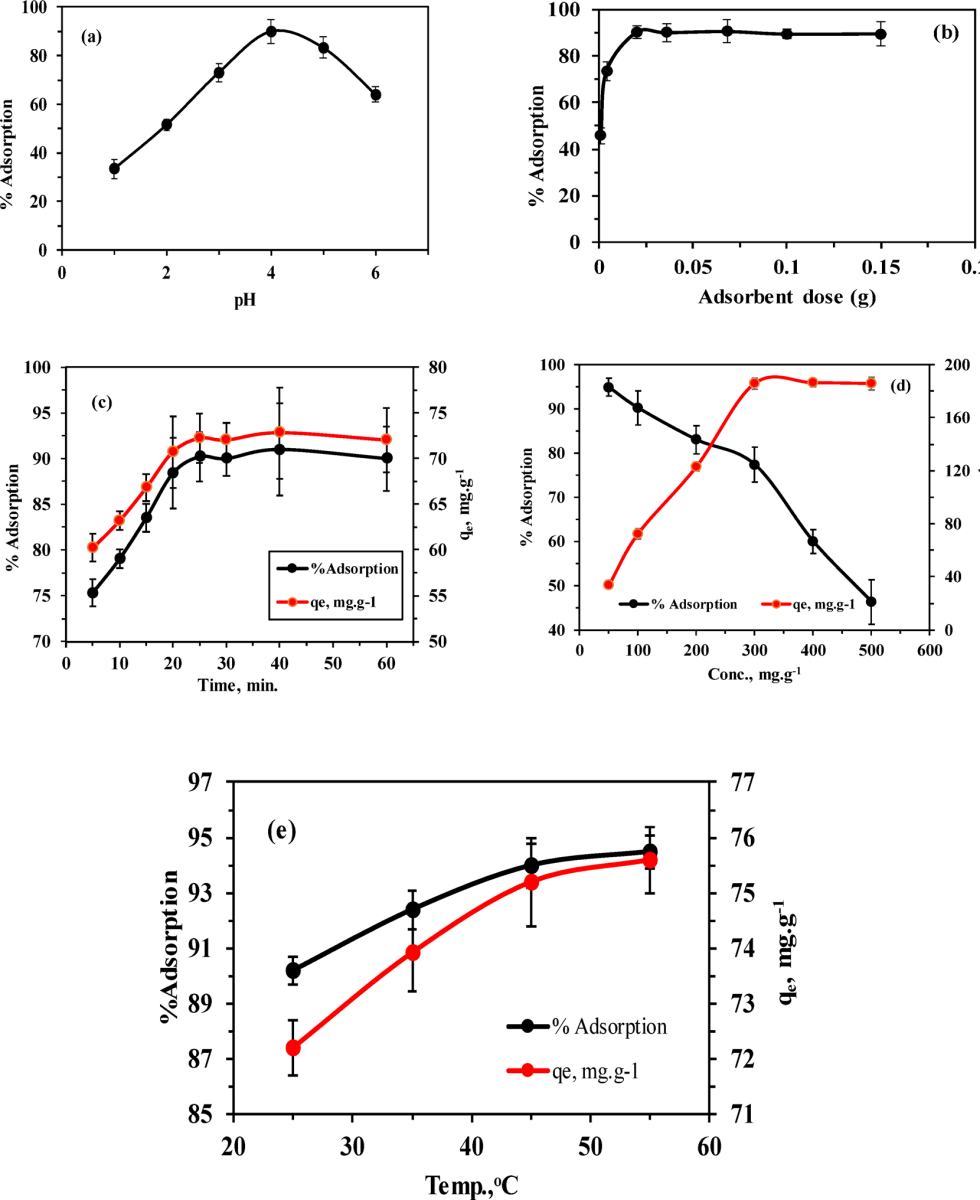
(**a**) Effect of pH (**b**) Effect of adsorbent dose, (**c**) Effect of contact time, (**d**) Effect of Th^4+^ initial concentration, (**e**) Effect of temp.

Several experiments were conducted to achieve the optimal pH and maximum adsorption efficiency of Th^4+^. The pH levels ranged from 1 to 6, with an initial Th^4+^ concentration of 100 mg L^−1^, an adsorbent dose of 1 g L^−1^, and a shaking time of 30 min. As shown in (Fig. 8a), as the pH increased from 1 to 4, the concentration of hydrogen ions that compete with Th^4+^ for adsorption sites decreased. This allowed for a gradual increase in Th^4+^ adsorption efficiency from 33.4% to 90.0%. The main functional group in MCC is the carboxyl group, which has a pKa value of about 3^61^. At pH levels below 3, the nonionic form of carboxylic acid is present as –COOH, leading to low Th adsorption. However, at pH levels above 3, the carboxyl group exists as –COO–, which enhances Th adsorption. The pH also affects the active sites of the adsorbent and the speciation and bio sorption availability of Th^4+^. Above pH 4, insoluble thorium hydroxide begins to precipitate as Th (OH)_2_^3+^, Th (OH)_2_^2+^, Th_2_(OH)_2_^6+^, and Th_6_(OH)_15_^9+^^34,36,62^, causing a decrease in adsorption efficiency. Generally, the optimal pH range for Th^4+^ adsorption is between 3 and 5 (Table 3). Several studies have reported optimal pH at around 3 for alkali-treated aloe vera wastes and modified pomegranate peel^7,8^, pH 4 for Ginkgo leaf, rice husk, and date seed^2,33,36^, pH 4.5 for citric acid-treated mangrove^62^ and pH 5 for citric acid-modified Lemna minor^34^. In this study, pH 4 was chosen as the optimum.

**Table 3:** maximum adsorption capacity of thorium onto different natural adsorbents.

Adsorbent	q_e_ (mg g^−1^)	pH	References
Citric acid treated mangrove endophytic fungus Fussarium sp.	75.47	4.5	Yang et al.^62^
Ginkgo leaf	103.8	4	Huang et al.^2^.
Alkali treated aloe vera wastes	170	3	Kapashi et al.^7^
Modified pomegranate peel	80	3	Noli, et al.^8^
Rice-husk	15.95	4	Varala et al.^33^
The citric acid modified Lemna minor	162.5	5	Yang et al.^34^
Date seed	43	4	Sayed et al.^36^

#### Effect of the adsorbent dose

To evaluate the effect of adsorbent dose, different doses of MCC we used, ranging from 0.001 to 0.15 g, with 20 ml of a 100 mg L^−1^ initial thorium concentration at pH 4 and a 30 min shaking time. As shown in (Fig. 8b), the adsorption efficiency of thorium increases gradually with the adsorbent dose, reaching a maximum at a certain point, and then leveling off. This is because increasing the adsorbent dose increases the surface area and the number of active sites, with an adsorption efficiency of 90% achieved at 0.02 g of adsorbent. However, beyond this point, all active sites on the adsorbent are occupied by thorium ions, and further increases in adsorbent dose have no noticeable effect^51,52^. Thus, the optimal adsorbent dose was selected to be 1 g L^−1^.

#### Effect of contact time

The effect of shaking contact time upon adsorption of Th^4+^ had been studied from 5 min to 60 min, adsorbent dose 0.02 g using 20 mL of 100 mg L^−1^ Th^4+^ solution, pH 4 and at room temperature. The study found that the equilibrium contact time is the point at which Th^4+^ adsorption onto MCC reaches its maximum capacity. As shown in (Fig. 8c), Th ions removal is relatively fast and increases with contact time, due to the availability of many active surface sites. Once equilibrium is reached, the adsorption efficiency remains steady. The highest adsorption efficiency achieved was 90.2% at 25 min, with an adsorption capacity of 72.2 mg g^−1^. Previous studies by Yang et al.^34^ and Sayed et al.^36^ reported equilibrium times of 20 min and 60 min, respectively.

#### Effect of initial concentration

Multiple experiments were carried out to examine how the initial concentration of thorium ions impacts its removal from a solution. The experiments utilized different Th^4+^ concentrations between 50 and 500 mg L^−1^, with a constant adsorbent dose of 0.02 g, a pH of 4, and a contact time of 25 min at room temperature. As illustrated in (Fig. 8d), the results show that adsorption efficiency dropped from 95% to 46.4% as the initial concentration rose from 50 to 500 mg L^−1^. Initially, MCC displayed high adsorption efficiency due to its numerous available binding sites, with a maximum adsorption capacity of 185.7 mg g^−1^ at an initial Th^4+^ concentration of 300 mg L^−1^. However, further increases in Th concentration had minimal impact on adsorption capacity, as the active binding sites on MCC reached saturation^34^.

#### Effect of adsorption temperature

Various experiments were run at temperatures between 25 °C and 55 °C to investigate how temperature affects Th^4+^ adsorption. Other variables were kept constant: 20 ml of 100 mg L^−1^ Th^4+^ solution with 0.02 g of MCC, pH 4, for 25 min. As shown in (Fig. 8e), the adsorption process showed slightly temperature dependence, with efficiency increasing from 90.2% to 94.5% as temperature rises from 25 °C to 55 °C. This suggests that the adsorption mechanism is endothermic. The adsorption capacity peaked at 75.6 mg g^−1^ at 55 °C. This finding is consistent with the work of Leyva et al.^27^, who used modified corncob with citric acid as an adsorbent for extracting Cd (II).

### Adsorption isotherms

Adsorption isotherm models, such as Langmuir and Freundlich, describe the relationship between equilibrium metal ion concentration in solution and the amount adsorbed (q_e_) under constant temperature and pH across varying initial concentrations^51,52^. The Langmuir isotherm^63^ is based on the assumption that a homogeneous surface has equivalent adsorption sites, forming a saturated monolayer of adsorbate. After the monolayer forms, the adsorption is less dependent on the adsorbate concentration. This model also assumes that all sites are energetically equivalent and that there are no significant interactions between the adsorbed species on the surface. The Langmuir isotherm can be represented mathematically as follows:

8 $$\frac{{{C_e}}}{{{q_e}}}=\frac{1}{{b~{q_{max}}~}}~+\frac{{{C_e}}}{{{q_{max}}}}$$

**Fig. 9:**
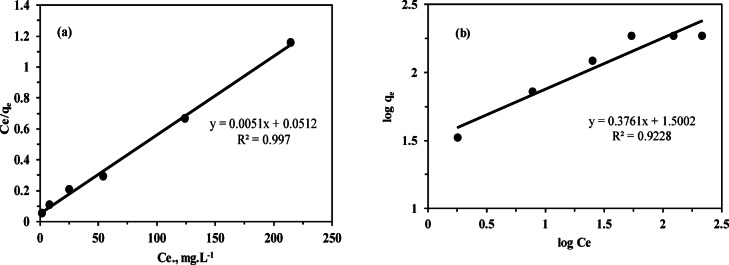
(**a**) Langmuir isotherm plot, (**b**) Freundlich isotherm plot for adsorption of Th^4^ upon MCC.

Where C_e_ (mg L^−1^) is the equilibrium concentration, q_e_ (mg g^−1^) is the amount of thorium adsorbed at equilibrium, and q_max_ (mg g^−1^), b (L.mg^−1^) are constants related to adsorption capacity and energy of adsorption, respectively. As shown in (Fig. 9a), plotting C_e_/q_e_ versus C_e_ yields a straight line. The values of q_max_ and b were determined from the slope and intercept of (Fig. 9a), which were found to be 196.08 mg g^−1^ and 0.098 L.mg^−1^, respectively.

The Langmuir isotherm model can be explained in terms of the dimensionless constant (R_L_) expressed as in the following equation:

9 $$R_{L}=\frac{1}{{1+b{C_0}}}$$

Here, b represents the Langmuir constant, and C_0_ is the initial thorium concentration. The separation factor (R_L_) offers insight into the adsorption process. There are four potential outcomes for the R_L_ value: when 0 < R_L_ < 1, adsorption is favorable; if R_L_ > 1, adsorption is unfavorable; if R_L_ = 1, it indicates linear adsorption; and if R_L_ = 0, adsorption is irreversible. In this study, the calculated R_L_ values from Eq. 9 fall between 0.02 and 0.17, indicating that the adsorption of Th^4+^ onto MCC is favorable.

According to the Freundlich isotherm model, the adsorbent surface is heterogeneous, with a non-uniform distribution of active sites that have different affinities for the adsorbate. This leads to multilayer adsorption, where interactions occur between molecules on the surface^64^. The Freundlich isotherm model can be expressed using the following equation.

10 $$logq{~_e}={\mathrm{log}}{{\mathrm{K}}_{{\mathrm{f}}}}+\frac{1}{n}\log {C_e}$$

Here, K_f_ (mg g^−1^) represents the adsorbent capacity, and 1/n represents the sorption intensity of the adsorbent. The Freundlich constants K_f_ and 1/n are determined from the slope and intercept of the log q_e_ versus log C_e_ plots, as shown in (Fig. 9b). A value of n between 1 and 10 indicates favorable adsorption. In this study, the K_f_ value was 31.64 mg g^−1^, indicating the adsorption affinity of MCC toward Th^4+^, while n was 2.66, suggesting favorable adsorption. Previous results as illustrated in (Table 4) showed that the Langmuir isotherm provides a better fit for the adsorption of Th^4+^ onto MCC, with a maximum adsorption capacity of 196.08 mg g^−1^ and a correlation coefficient of 0.997, which is higher than that obtained from the Freundlich model (R^2^ = 0.9228).

**Table 4:** Isotherm models constants for Th^4+^ adsorption onto MCC.

Model	Parameter	Value
Langmuir	q_max_(exp)	185.7 mg g^−1^
	q_max_ (calc)	196.08 mg g^−1^
	b	0.098 L mg^−1^
	R_L_	0.02–0.17
	R^2^	0.997
Freundlich	K_f_	31.64 mg g^−1^
	n	2.66
	R^2^	0.9228

### Adsorption kinetics

Several adsorption kinetic models have been used to understand the mechanism that control the adsorption process. In this study Lagergren pseudo-first order^65^ and Ho’s pseudo-second order^66^ models were employed to investigate the adsorption of Th^4+^on MCC, and their rate equations were represented respectively as following:

11 $$\log (~{q_{e - ~~~}}{q_{t~~}})=log{q_{e~~}} - \left( {\frac{{{k_1}}}{{2.303}}} \right)t$$

12 $$\frac{t}{{{q_t}}}=~\frac{1}{{{k_{2~~}}q_{e}^{2}}}+\left( {\frac{1}{{{q_e}}}} \right)t~$$

**Fig. 10:**
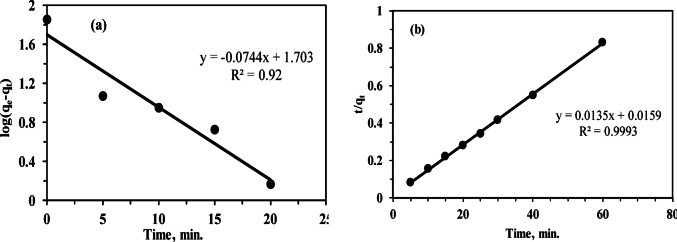
(**a**) Pseudo- first order kinetic plots and (**b**) Pseudo-second order kinetic plots for the adsorption of Th (IV) on MCC.

Here, q_t_ (mg g^−1^) and q_e_ (mg g^−1^) represent the adsorption capacities at time t (min) and at equilibrium, respectively. k_1_ (min^–1^) and k_2_ (g mg^–1^ min^–1^) are the rate constants for the pseudo-first-order and pseudo-second-order models, respectively. The pseudo-first-order kinetic model implies that Th^4+^ adsorption on MCC involves weak interactions, whereas the pseudo-second-order model assumes the adsorption mechanism is controlled by chemical reactions, making the adsorption efficiency of Th^4+^ directly proportional to the available active sites on MCC. From the linear plots of log(q_e_ − q_t_) versus t and t/q_t_ versus t in (Fig. 10a,b), the theoretical q_e_ values from the first-order kinetic model are lower (50.46 mg g^−1^) compared to the experimental values (72.2 mg g^−1^), with a correlation coefficient R^2^ of 0.9255. In contrast, the theoretical q_e_ values from the pseudo-second-order kinetic model are 74.07 mg g^−1^, closely matching the experimental data, with a correlation coefficient R^2^ of 0.9993. The reaction rate constants for both models are shown in (Table 5). Therefore, the adsorption process aligns well with the pseudo-second-order model and is controlled by chemisorption, whereby Th^4+^ binds to the active sites of MCC through chemical bonds^49^.

**Table 5:** The rate reactions values for adsorption of thorium onto MCC.

Model	Parameters	Value
	q_e_ (exp)	72.2 mg g^−1^
Pseudo-first order	K_1_	0.171 min^−1^
	q_e_ (calc)	50.46 mg g^−1^
	R^2^	0.9255
Pseudo-second order	K_2_	0.011 g mg^−1^ min^−1^
	q_e_ (calc)	74.07 mg g^−1^
	R^2^	0.9993

### Thermodynamic studies

Thermodynamics studies were performed to evaluate the feasibility and nature of the adsorption process. The thermodynamic parameters including Gibbs free energy △G (kJ mol^−1^), enthalpy ΔH (kJ mol^−1^), and entropy change ΔS (J mol^−1^ K^−1^) were determined to describe the adsorption behavior^67^. The Gibbs free energy change can be estimated using classical Van’t Hoff Eq. (14) at different temperatures 298, 308, 318 and 328 k respectively.

13 $$\Delta G= - {\mathrm{RT}}\ln {{\mathrm{k}}_{\mathrm{D}}}$$

Gibbs free energy was also related to enthalpy and entropy at constant temperature according to following equations. 

14 $$\Delta {\mathrm{G}}={\mathrm{DH}}-{\mathrm{TDS}}$$

The previous two relations give the following Eq. (15)

15 $${\text{Ln }}{{\mathrm{k}}_{\mathrm{D}}}={\mathrm{DS}}/{\mathrm{R}}-{\mathrm{DH}}/{\mathrm{RT}}$$

**Fig. 11:**
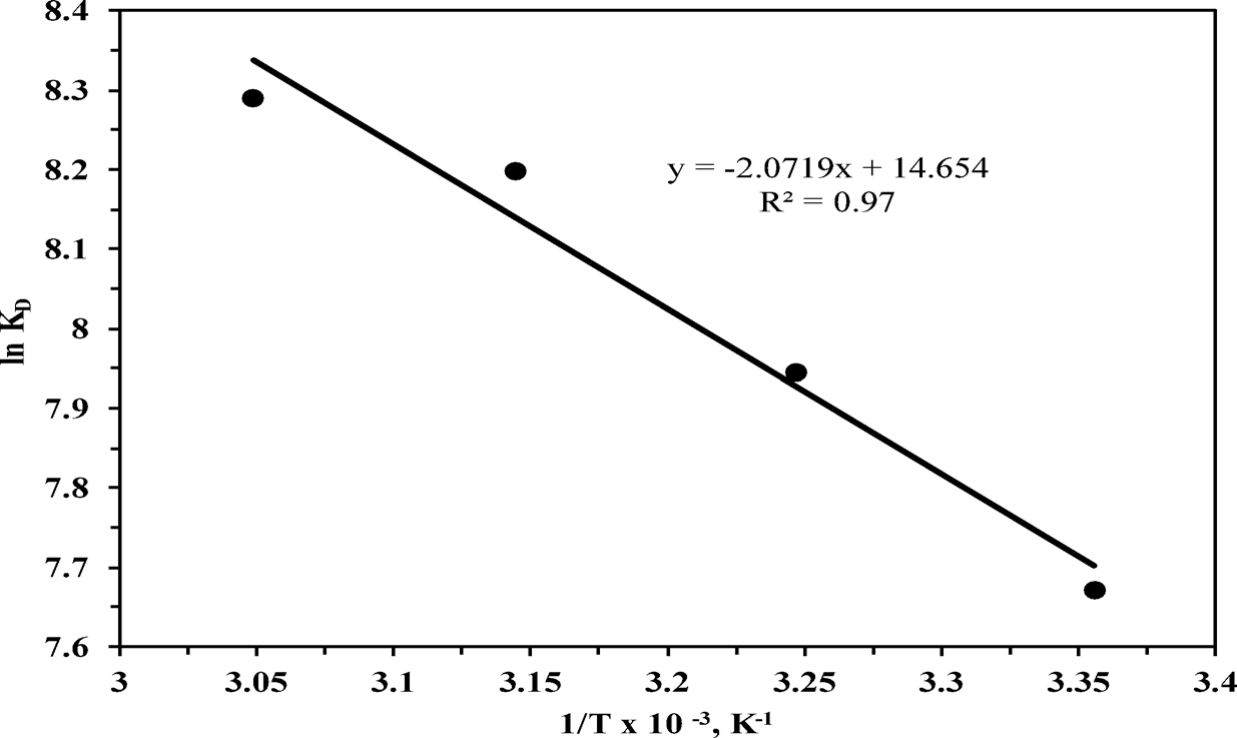
A plot of ln k_D_ verses 1/T x10^− 3^1000/T for thorium adsorption.

Where T is the absolute temperature (K), R is the universal gas constant (8.314 J mol^−1^ K^−1^), and k_D_ is the equilibrium constant. Based on the results from plotting ln k_D_ versus 1000/T (Fig. 11), which shows a straight line with a correlation coefficient of 0.9702, the slope equals –ΔH/R and is used to calculate ΔH, while the intercept equals ΔS/R and is used to calculate ΔS. These ΔH and ΔS values, along with Eq. (14), are used to calculate △G, as shown in (Table 6). The negative Gibbs free energy △G obtained at all temperatures indicates that the adsorption process is spontaneous and favorable^39^. The enthalpy change ΔH for the adsorption reaction is 17.23 kJ.mol^−1^, with a positive value confirming the endothermic nature of Th^4+^ adsorption by MCC. The positive entropy ΔS (121.83 J.mol^−1^ K^−1^) reflects increased randomness at the solid-liquid interface during the adsorption process. The calculated values of TΔS at different temperatures as listed in (Table 6) and increase with increasing temperature due to the direct proportionality between temperature and entropy, indicating an enhanced entropy contribution to the Gibbs free energy at higher temperatures.

**Table 6:** Thermodynamic parameters for adsorption of Th^4+^ onto MCC.

T, K	ΔH (kJ mol^−1^)	ΔS (J mol^−1^K^−1^)	TΔS (J mol^−1^)	ΔG (kJ.mol^−1^)	*R* ^2^
298	17.23	121.83	36305.34	− 19.08	0.9702
308			37523.64	− 20.298	
318			38741.94	− 21.517	
328			39960.24	− 22.735	

### Optimization of elution process

The loaded adsorbent with thorium was prepared by applying the optimal adsorption conditions on 0.075 g of MCC with 75 ml of 300 mg L^−1^ Th^4+^ solution, and shaken for 25 min at room temperature.

#### Effect of eluting agent type

**Fig. 12:**
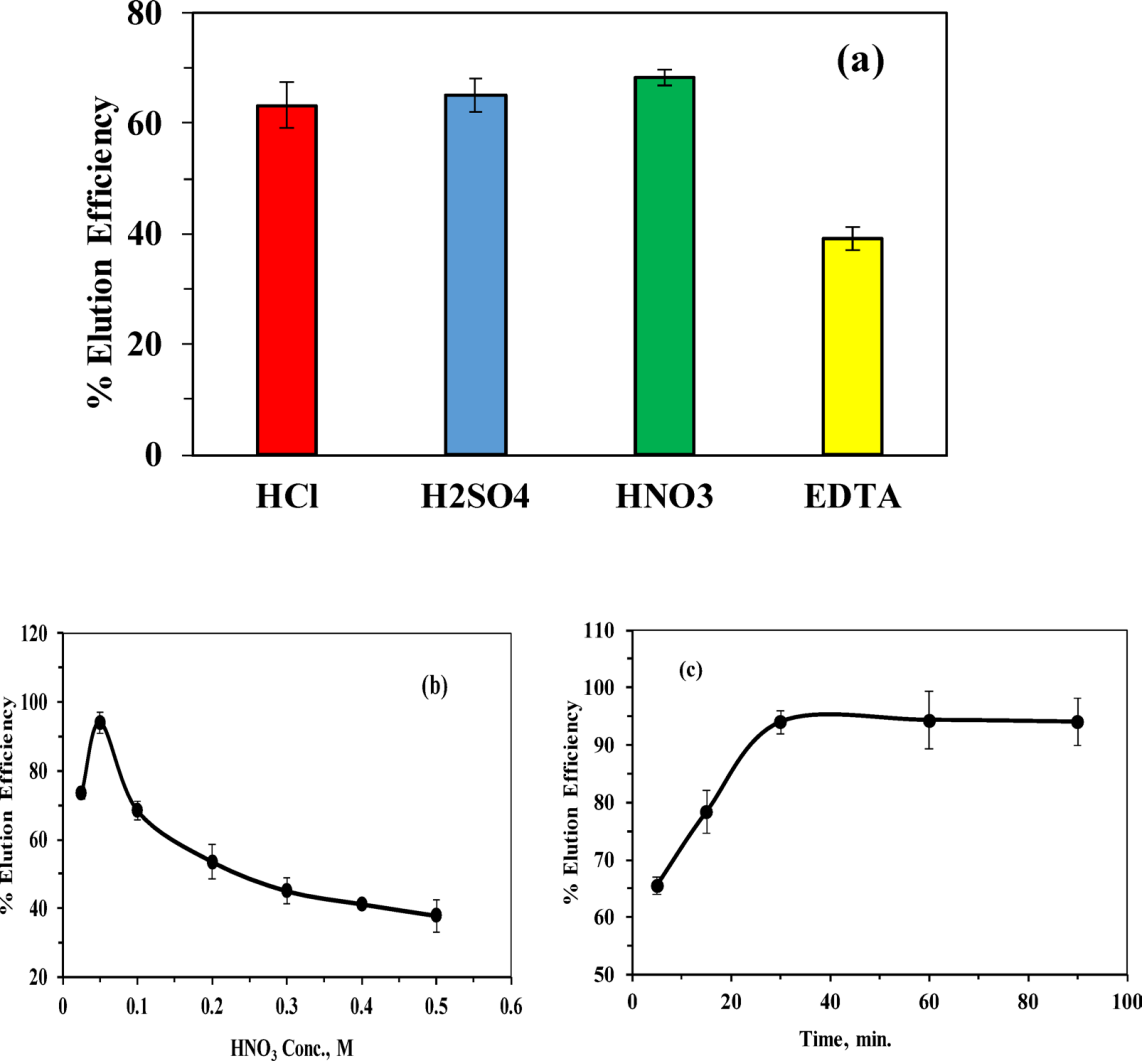
(**a**) Effect of eluting agent type, (**b**) Effect of eluting agent conc., (**c**) Effect of eluting time.

The effect of the eluting agent type on thorium desorption was investigated by shaking 0.005 g of Th^4+^-loaded adsorbent with 5 mL of 0.1 M HCl, HNO_3_, H_2_SO_4_, and EDTA for 30 min at room temperature. As illustrated in (Fig. 12a), nitric acid exhibited the highest elution efficiency attained to 68.31% compared with the other eluents, similar results using HNO_3_ as the eluent with different desorption efficiencies, have been reported in the literature^36,68^. Although HCl and H_2_SO_4_ are also capable of forming soluble thorium species through protonation and ligand interaction, the superior performance of HNO_3_ is may be attributed to the formation of more stable and mobile thorium–nitrate complexes. In contrast, chelating agents such as EDTA rely predominantly on complexation mechanisms, which may be kinetically limited under short contact times. Accordingly, nitric acid was chosen as the optimal eluent for thorium desorption.

#### Effect of eluting agent concentration

The influence of nitric acid concentration on the elution efficiency was studied over the range of 0.025–0.5 M using 0.005 g of adsorbent and a contact time of 30 min at room temperature. As shown in Fig. 12b, increasing the HNO_3_ concentration from 0.025 to 0.05 M significantly enhanced the elution efficiency from 73.5% to 94%. This improvement is attributed to the increased availability of H^+^ ions, which effectively protonate the citric-acid-derived carboxylate groups (–COO^−^), weakening the electrostatic and coordination interactions with Th^4+^ and promoting its release into the solution. However, a further increase in nitric acid concentration resulted in a noticeable decline in elution efficiency. At higher acid strengths, the MCC surface becomes highly protonated, acquiring a net positive charge. Simultaneously, thorium undergoes speciation changes in nitrate-rich media, forming neutral and negatively charged complexes such as [Th(NO_3_)_4_], [Th(NO_3_)_5_]⁻, and [Th(NO_3_)_6_]^2^⁻. The electrostatic attraction between these anionic thorium species and the positively charged surface promotes partial re-adsorption of thorium, thereby reducing the overall elution efficiency. This behavior is consistent with thorium nitrate speciation studies^69^ and previous findings in actinide separation chemistry^70^. Consequently, 0.05 M HNO_3_ was selected as the optimal eluent concentration.

#### Effect of eluting agent contact time

The effect of contact time on thorium elution was evaluated over a period ranging from 5 to 90 min using 0.05 M HNO_3_ at room temperature. As depicted in (Fig. 12c), the elution efficiency increased rapidly from 65.5% at 5 min to 94% at 30 min, indicating fast desorption kinetics and efficient disruption of Th–adsorbent interactions during the initial stage. Beyond 30 min, no significant improvement in elution efficiency was observed, suggesting that desorption equilibrium had been reached and that most accessible binding sites had already been regenerated. Therefore, a contact time of 30 min was considered sufficient and optimal for effective thorium elution.

## Regeneration studies

The reusability of an adsorbent plays a key role in determining its overall performance and practical applicability. Desorption of Th^4+^ ions from aqueous solution was carried out under the optimum elution conditions. After each desorption cycle, the MCC was filtered, dried, and reused in subsequent adsorption experiments. The results illustrated in (Table 7) indicate that after five consecutive cycles, the adsorption efficiency of Th^4+^ on MCC decreased slightly from 90.2% to 84%, while the elution efficiency using 0.05 M HNO_3_ declined from 94% to 88%. These results demonstrate that MCC can be effectively regenerated and reused. The good regeneration performance is attributed to the chemical stability of the citric-acid-modified functional groups on the corncob surface, which remain active after repeated protonation and desorption. Overall, the adsorbent exhibits both high efficiency and good reusability, making it a promising candidate for sustainable thorium recovery from aqueous solutions.

**Table 7:** Thorium adsorption–desorption and adsorbent recycling.

Cycle No.	Adsorption (%)	Desorption (%)
1	90.2	94
2	89.3	93.5
3	87.9	91.8
4	86.0	90.0
5	84.0	88

## Application

**Fig. 13:**
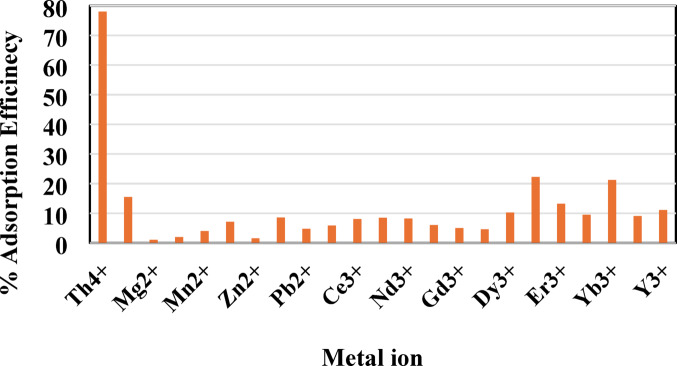
The adsorption efficiency of thorium and the other metal ions from the leached solution.

The presence of coexisting ions in the leaching solution of the studied sample has a significantly impact the adsorption behavior of thorium by competing for active sites on MCC. The metal ions concentrations in the leachate before and after adsorption were determined and presented in (Table 8). The results indicate that the adsorption efficiency of thorium decreased to reach 78% compared to single thorium solution and moderate adsorptivity of 22. 22%, 21.26% and 15.48% were obtained for Ho^3+,^ Yb^3+^ and UO_2_^2+^ respectively. whereas other metals exhibited significantly lower adsorption rates. The MCC exhibited the highest selectivity for Th, likely due to the tetravalent nature of Th^4+^, which generates a stronger electrostatic attraction to the carbonyl and hydroxyl groups compared to trivalent cations^2^. Figure 13 shows the effectiveness of MCC as an adsorbent for removing thorium from leachate in the presence of competing ions.

**Table 8:** Concentration of elements before and after adsorption.

Metal ion	Conc. of metal ion before adsorption, mg L^−1^	Conc. of metal ion after adsorption, mg L^−1^	Adsorption efficiency, %
Th^4+^	175	38.5	78
UO_2_^2+^	61	51.55	15.48
Mg^2+^	2.83	2.8	1.06
Ca^2+^	13.36	13.1	1.95
Mn^2+^	1.01	0.97	3.96
Fe^3+^	0.28	0.26	7.14
Zn^2+^	7.71	7.59	1.56
Al^3+^	5785.2	5290.3	8.55
Pb^2+^	7.77	7.40	4.76
La^3+^	1.36	1.28	5.88
Ce^3+^	6.75	6.21	8.0
Pr^3+^	1.42	1.30	8.45
Nd^3+^	1.34	1.23	8.21
Sm^3+^	0.40	0.34	6.0
Gd^3+^	0.45	0.40	5.0
Tb^3+^	0.22	0.21	4.54
Dy^3+^	0.98	0.88	10.2
Ho^3+^	0.09	0.07	22.22
Er^3+^	0.38	0.33	13.16
Tm^3+^	0.21	0.19	9.52
Yb^3+^	0.254	0.20	21.26
Lu^3+^	0.11	0.10	9.09
Y^3+^	3.07	2.73	11.07

## Conclusion

Studies have shown that citric acid-modified corncob (MCC) is a highly effective, eco-friendly, and affordable material for removing thorium from aqueous solution by adding extra carboxylic groups through chemical modification, the surface acidity, porosity, and surface area increased significantly, as confirmed by various analysis techniques. The research found that the adsorption process reached equilibrium within 25 min and achieved a maximum efficiency of 90.2% at pH 4, with a Langmuir monolayer adsorption capacity of 196.08 mg g^−1^. This suggests a strong attraction between Th^4+^ ions and the modified surface. Kinetic and thermodynamic tests revealed that the adsorption process followed the pseudo-second-order model, driven by chemisorption, and was both spontaneous and endothermic. Additionally, desorption experiments showed that 0.05 M HNO_3_ effectively regenerated the adsorbent, maintaining over 84% efficiency after five cycles, which highlights its stability and reusability. Overall, the results confirm that citric acid modified corn cob is a promising, sustainable, and efficient material for thorium removal and recovery from aqueous systems.

## Data Availability

All data generated or analyzed during this study are included within the article.
